# How to Optimize the Use of Blood Cultures for the Diagnosis of Bloodstream Infections? A State-of-the Art

**DOI:** 10.3389/fmicb.2016.00697

**Published:** 2016-05-12

**Authors:** Brigitte Lamy, Sylvie Dargère, Maiken C. Arendrup, Jean-Jacques Parienti, Pierre Tattevin

**Affiliations:** ^1^Laboratoire de Bactériologie, CHU MontpellierMontpellier, France; ^2^Service de Maladies Infectieuses, CHU CaenCaen, France; ^3^Unit for Mycology, Department of Microbiology & Infection Control, Statens Serum InstitutCopenhagen, Denmark; ^4^Unité de Biostatistiques et de Recherche Clinique, CHU CaenCaen, France; ^5^Service de Maladies Infectieuses, CHU RennesRennes, France

**Keywords:** bloodstream infection, blood culture, single sampling strategy, multi-sampling strategy, sensitivity, specificity, contamination

## Abstract

Bloodstream infection (BSI) is a major cause of death in developed countries and the detection of microorganisms is essential in managing patients. Despite major progress has been made to improve identification of microorganisms, blood culture (BC) remains the gold standard and the first line tool for detecting BSIs. Consensus guidelines are available to ensure optimal BSI procedures, but BC practices often deviate from the recommendations. This review provides an update on clinical and technical issues related to blood collection and to BC performance, with a special focus on the blood sample strategy to optimize the sensitivity and specificity of BCs.

## Introduction

Bloodstream infections (BSIs) represent a growing public health concern, with an estimated burden of 1,200,000 episodes of BSI each year in Europe, and 157,000 attributable deaths (Goto and Al Hasan, 2013). BSI ranks among the top seven causes of death in North America and Europe. The diagnosis of BSI relies on the documentation of pathogens in blood—either bacteremia, or fungemia—through blood cultures (BCs). Indeed, although the last decades have experienced dramatic achievements in the development of rapid diagnostic tests relying on innovative technologies, BCs remain the gold-standard not only for the diagnosis of BSI, but also for the identification of the responsible pathogen(s), and for the testing of their susceptibility to anti-infective agents. Since the mid-1970s, various original studies, systematic reviews, and guidelines to better define the principles and practices of BCs sampling and processing have been published. The available evidence suggests that the diagnostic yield of BCs is influenced by the collection of appropriate specimens, from selected patients with reasonable suspicion of BSI. We performed a systematic literature review on clinical and technical issues related to blood collection, as well as interpretation of BCs, in adult patients suspected of BSI. We focused on the impact of BC collection strategies on their performance for the diagnosis of BSI, as this has not been a major focus in most recent reviews (e.g., Kirn and Weinstein, 2013; Garcia et al., 2015).

## Ordering blood cultures

Published guidelines do not clearly state when BCs should be ordered (Baron et al., 2013). Blood cultures are commonly collected when patients have fever, chills, leukocytosis, septic shock, suspected endocarditis or prior to starting antimicrobial treatment in elderly or immunocompromised patients.

Physicians significantly overestimate the likelihood of BSI for their patients (Poses and Anthony, 1991). Indeed, in most settings, only 5 to 13% of BCs will turn out to be positive, and of those, 20–56% represent contaminants (Bates et al., 1990; Salluzzo and Reilly, 1991; Little et al., 1997; Dargère et al., 2014). Despite progress in skin antisepsis since 25 years led to lower risk of contamination (e.g., 0.5–1%, Garcia et al., 2015), rates of contamination as high as 2.1–6% are still commonly reported, and the 20–56% proportion of contaminants holds true today (e.g., Zwang and Albert, 2006; Gander et al., 2009; Roth et al., 2010; Dargère et al., 2014).

Many models for predicting bacteremia have been developed but not all were validated and, when they were, the validation processes were highly heterogeneous (Bates et al., 1990; Shapiro et al., 2008; Coburn et al., 2012). Eliakim-Raz et al. (2015) identified studies that underwent validation on prediction of bacteremia and that were able to define groups with low (<3%) or high (>30%) probabilities of bacteremia in adults. They demonstrated that few studies have been prospectively validated, populations and parameters included were heterogeneous and none of these models were implemented in clinical practice (Eliakim-Raz et al., 2015). The additional workload required to enter data may explain the reluctance to use these models. Multicenter clinical trials would be necessary to better define the added value of models to guide BC ordering strategies in clinical practice.

## Timing of BC specimen collection

Thus far, the most appropriate timing of BC collection has been poorly evaluated through clinical studies. Most guidelines state that blood specimens should be collected in the absence of antimicrobials, at or around the time of fever spikes, and a 30–60 min interval between samples has been arbitrarily recommended (Weinstein, 1996). However, in a seven-center study that evaluated the timing of BC collection in relation to body temperature in 1436 patients with BSI, Riedel et al. (2008) could not identify any optimal timing for BC collection. Indeed, the likelihood of documenting BSIs was not significantly enhanced by collecting blood during temperature spikes. Yields were similar over a 24-h period before and after temperature spikes (Riedel et al., 2008). Consistent with these results, Li et al. (1994) found no difference in BCs yield whether samples were collected within a 24-h period, either simultaneously or serially (Li et al., 1994).

## Blood culture collection

### Skin preparation

Meticulous skin antisepsis at the time of blood collection is paramount to reduce the risk of BC contamination, so that the rate of contaminants would remain below the threshold of 3% of all BCs sampled (Garcia et al., 2015). In a systematic review, Malani et al. (2007) found that: (i) alcoholic iodine tincture is more effective than aqueous povidone-iodine (PVI) to reduce the risk of BC contamination; (ii) alcoholic chlorhexidine gluconate (CHG) is more effective than aqueous PVI; (iii) alcoholic antiseptics are more effective than aqueous products, and (iv) that alcohol alone is not inferior to any iodine products (Malani et al., 2007). In a more recent meta-analysis of 6 clinical trials, alcoholic chlorhexidine was found to be more effective than non-alcoholic PVI, while alcoholic solutions were more effective than non-alcoholic solutions. This review found no significant difference between chlorhexidine and iodine products for skin antisepsis before blood collection (Caldeira et al., 2011). The Clinical and Laboratory Standards Institute (CLSI) guidelines for BCs collection favor the use of CHG for infants >2 months of age, children and adults (Wilson et al., 2007); and the Food and Drug administration recommended to use it with care in premature infants or infants under 2 months of age. These products may cause irritation or chemical burns (Schiffenbauer, 2012).

A recent randomized crossover trial comparing the effectiveness of 3 skin antiseptic interventions—10% aqueous PVI solution, 2% iodine tincture (IT), and 2% CHG in 70% isopropyl alcohol—before BCs sampling, found that the choice of antiseptic agent does not impact contamination rates when BCs are sampled by a dedicated phlebotomy team (Washer et al., 2013). A systematic review and meta-analysis concluded that the implementation of dedicated phlebotomy teams reduce the rate of BCs, while no evidence support the use of prepackaged prep kits (Snyder et al., 2012). In addition, it has been demonstrated that BCs contamination rates are significantly lower when an antiseptic agent is applied on BC bottle tops before sampling (Schifman et al., 1998). European and French guidelines recommend the use of an alcoholic solution for antisepsis before BC sampling (Lamy and Seifert, 2012; Accoceberry et al., 2015a).

For an extensive review on skin antisepsis before BC collection and prepackaged kits performance, see Garcia et al. (2015).

### Sampling site

Several studies concluded that peripheral venipuncture is the method of choice for BC collection, as compared with sampling through intravenous catheter, with rates of contamination ranging from 1.2 to 7.3% when samples are obtained from venipuncture compared with 3.4 to 13% when blood is drawn through catheter (Dawson, 2014; Garcia et al., 2015). Indeed, both colonization of the catheter, and breakdowns in sterile procedure increase the risk of BCs contamination when BCs samples are drawn through these devices (Bates et al., 1991). A multi-interventional study that included education of healthcare workers to avoid the sampling of BCs from central intravenous lines, documented simultaneous decreases in (i) the proportion of BCs obtained from central lines (from 10.9 to 0.4%); and (ii) BCs contamination rates (from 1.6 to 0.5%; Boyce et al., 2013). Although studies have shown that contamination rates are lower for BCs drawn from newly inserted catheter using a sterile technique protocol (Levin et al., 2013), BCs are usually even less contaminated when samples are drawn by peripheral venipuncture (Snyder et al., 2012).

One exception must be outlined: for the diagnosis of central line associated BSI (CLABSI), most current guidelines recommend simultaneous sampling of BC drawn from the suspected catheter, and through a venipuncture, to be able to estimate the differential time to positive BC (Baron et al., 2013; Dellinger et al., 2013). Indeed, if BC drawn from the central line grows at least 2 h earlier than BC drawn from venipuncture, the central line is most likely the source of BSI (Kirn and Weinstein, 2013). However, improper collection of BCs is associated with potential over-reporting of CLABSI (Kaasch et al., 2014; Garcia et al., 2015).

Despite concordant recommendations, the techniques for collection of BCs vary across countries in routine practice. A study performed in intensive care units across 4 European countries found that the preferred means for BCs collection were peripheral venipuncture in Germany and Italy (42 and 76% via peripheral venipuncture, respectively, vs. 8 and 0% via intravenous catheter, while 50 and 24% reported no preference), while sampling through intravenous catheter was more common in France and the UK (respectively, 33 and 23% via intravenous catheter, vs. 20 and 23% via peripheral venipuncture, while 47 and 54% reported no preference; Schmitz et al., 2013).

### Impact of volume sampled on BCs yield

Data available could be summarized as follows: “The higher the blood volume cultured, the higher the yield.” Indeed, adequate volume sampling is the most important parameter for the detection of bloodstream microorganisms because bacterial or fungal density in blood is very low in most patients with BSI. Basically, the likelihood of detecting a BSI depends on the bacterial or fungal concentration, and on the volume collected. Using theoretical models, Arpi et al. (1989) estimated the average bacterial concentration in patients with bacterial BSI at 0.25 colony-forming unit (CFU) per milliliter, and Jonsson et al. (1993) demonstrated that the bacterial concentration was less than 0.04 CFU/mL in 29% of *Escherichia coli* BSI and 18% of *Staphylococcus aureus* BSI (Arpi et al., 1989; Jonsson et al., 1993). The sensitivity of BC was estimated to be 95% when 3 CFU are sampled, which implies that at least 30 mL of blood are incubated (Jonsson et al., 1993). The results of this model are in full agreement with Washington's empirical data obtained 18 years earlier, showing that a total volume of at least 30 ml of blood is required for detecting 99% of BSI (Washington, 1975). There are very few clinical studies directly evaluating the bacterial concentration in BSI, because this measurement requires the use of quantitative BC techniques, which are particularly labor-intensive, and not routinely used. These studies are consequently either originating from the early years of BCs, decades ago (with potential limitation due to the quality of the culture media used), or more recent, based on the lysis-centrifugation technique, but limited to a specific type of BSI (Tables 1, 2). For instance, Wain et al. (1998) showed that 53% of 349 patients with enteric fever had a *Salmonella enterica* serovar Typhi concentration between 0.01 and 1 CFU/mL of blood (Wain et al., 1998). In Reynes's study, 54.3% of 1026 patients with BSI (any type) had a bacterial density between 0.1 and 2 CFU/ mL of blood (Reynes, 1947). Similar data have been observed with *Candida* BSI (Table 2), and in Pfeiffer et al.'s study, 53% of 152 patients had a fungal density of less than 1 CFU/mL (Pfeiffer et al., 2011). Overall, data from modeling, as well as clinical studies are remarkably concordant: 50% of BSI episodes are associated with a bacterial concentration in the range of 0.01–1 CFU/mL (Tables 1, 2).

**Table 1:**
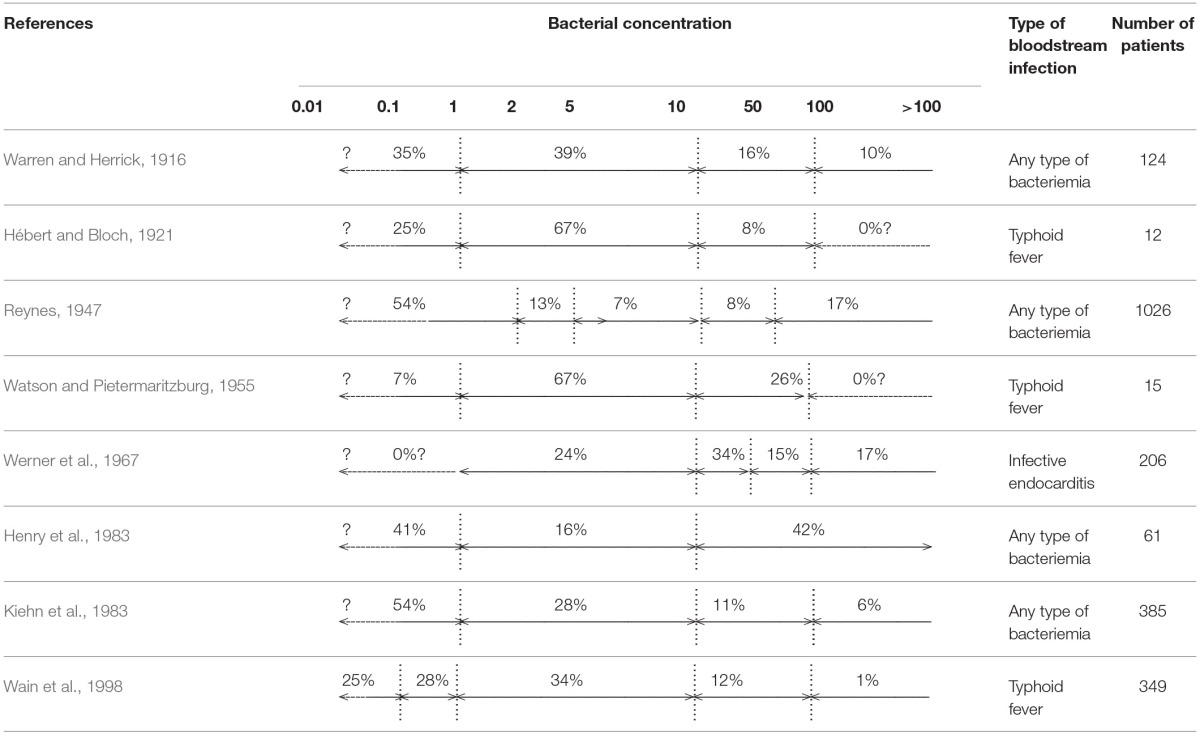
**Bacterial concentration in blood from adult bloodstream infections**.

All studies from this table performed colony count using the pour plate or spread plate technique. As methodology varies between studies, concentration range and categories vary. Of note, the quality and fertility of culture media may have varied between 1916 and 1998.

**Table 2:**
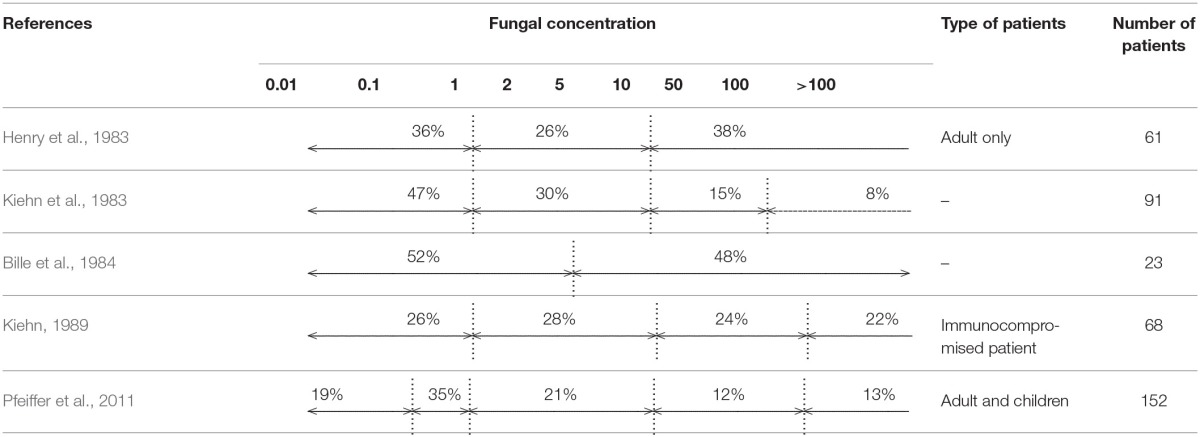
**Fungal concentration in blood from bloodstream infection**.

Comparing the yield of standard-volume BC (mean, 8.7 mL), and low-volume BC (mean, 2.7 mL), a study from the Wisconsin Hospital and Clinics demonstrated that the sensitivity of BCs for the diagnosis of BSI was 92% with standard-volume, and 69% with low-volume (difference of 23%, [95% CI, 9–37%]; *P* < 0, 001; Mermel and Maki, 1993). Examining 7783 BCs, including 624 classified as true positive BCs, Li et al. (1994) demonstrated that increasing the volume of blood cultured from 20 to 40 mL increased the yield by 19%, with an additional gain of 10% when the incubated volume was increased from 40 to 60 mL (Li et al., 1994). Since then, several studies have confirmed that the volume of blood cultured is the key parameter of BC yield (Cockerill et al., 2004; Patel et al., 2011). The most recent studies showed that sampling blood volumes of 20, 40, and 60 mL was associated with sensitivities of 65.0–75.7%, 80.4–89.2%, and 95.7–97.7%, respectively (Cockerill et al., 2004; Bouza et al., 2007; Lee et al., 2007; Patel et al., 2011).

Of note, since Washington's seminal works (Washington, 1975), the recommended volume of blood to be cultured gradually increased over decades. This increase resulted both from the need to develop (and accept) the concept of culturing larger volumes of blood, and from the study design on which data relied on: in the absence of perfect gold standard for detecting BSI, the 100% of BSI detected in each study is bounded by the maximum volume of blood cultured, with an inherent risk to under-detect BSI in case of lower volumes. Finally, since the 1970's, the changes in BSI epidemiology that have been reported may have been in part impacted by these changes in sampling techniques, and culture media system: as most BSI are associated with a low bacterial or fungal density, improvement in BCs sensitivity most likely increased the incidence of documented BSI, and may also have impacted on their patterns.

#### Current guidelines

As they are based on similar literature data, guidelines from scientific societies are in agreement on most issues. The UK Standards for Microbiology Investigations recommend 4 bottles (2 sets), corresponding to 20–30 mL per set (Public Health England, 2014). The French Society of Microbiology recommends that, in patients suspected of BSI, 4–6 bottles (2 to 3 sets) of blood should be cultured, with adequate volume for each bottle. A 6 bottles-procedure is necessary whenever the optimal filling of all the bottles is not ensured (Accoceberry et al., 2015a). The European guidelines also recommend the culture of 4–6 bottles adequately filled (Lamy and Seifert, 2012). The American College of Critical Care Medicine (ACCM) and Infectious Diseases Society of America (IDSA) Guidelines recommend that new fever in critically ill adult patients must be investigated by drawing of 3–4 BC sets with appropriate volume within the 24 h of fever onset (O'Grady et al., 2008). Guidelines from the American Society of Microbiology (ASM) have long been similar (Baron et al., 2005b). Recent ASM/IDSA joint guidelines for adults recommend 20–30 mL of blood per culture set, which, according to these guidelines, may require more than 2 bottles depending on the system. Generally, in adults with a suspicion of BSI, 2–4 BC sets should be obtained in the evaluation of each septic episode (Baron et al., 2013; Dellinger et al., 2013).

For optimal recovery, each BC set should include paired aerobic and anaerobic bottles, the aerobic bottle being filled first. Besides, the culture of only 2 bottles (1 set) during a 24-h period from adult patients (hereafter referred to as “solitary BC”), is discouraged in all guidelines, as the sensitivity of only 2 bottles is insufficient. In addition, it must be taken into account that, in the real life, a significant proportion of BCs bottles are not adequately filled (Vitrat-Hincky et al., 2011; Willems et al., 2012; Lin et al., 2013; van Ingen et al., 2013; Coorevits and Van den Abeele, 2015). The “solitary BC” practice, still common, is not acceptable, due to the detrimental outcome when BSI is not detected and appropriately managed, in terms of antimicrobial treatment (a positive BC will impact on the selection of active anti-infective agents for the appropriate treatment duration), but also to trigger the identification, and eradication of the BSI source (Lamy and Seifert, 2012; Baron et al., 2013; Accoceberry et al., 2015a).

#### Compliance with guidelines and current caveats

An increasing amount of data highlights the pre-analytical deficiencies of BC sampling which may compromise patient management by reducing BCs diagnostic yield (Schifman et al., 1991; Washington, 1992; Novis et al., 2001).

Firstly, BC bottles that should contain 8–10 mL of blood (manufacturer instructions) are frequently under-filled, consequently leading to sets of much less than 20 mL, or—although less commonly—over-filled (>10 mL). Mermel and Maki (1993) first highlighted the issue of under-filled BC bottles, a situation that was not center-specific, since 88% of 71 U.S. laboratories acknowledged that they routinely receive BC specimens from adults containing less than 5 mL of blood (Mermel and Maki, 1993). In Spain, in a prospective analysis conducted from 601 patients with suspected BSI, the mean volume of blood per BC bottle was estimated at 5 mL (Bouza et al., 2007). Similar sobering results have been reported from all over the world, with wide variations between countries (Table 3).

**Table 3:** **Quality of bottle filling**.

	**Under-filled bottles**		**Over-filled bottles**		
**References**	**Threshold (mL)**	**Rate (%)**	**Threshold (mL)**	**Rate (%)**	**Country**
Vitrat-Hincky et al., 2011	< 8	65	>10	13.0	France
Willems et al., 2012^a^^,^^b^	< 8	26.2–36.0	>12	7.6-12.8	Belgium
van Ingen et al., 2013	< 8	55.3	–	–	The Nederlands
Coorevits and Van den Abeele, 2015	< 8	28.0	>12	23.2	Belgium
Chang et al., 2015	< 8	97.7	>10	0.2	South Korea
Lin et al., 2013	< 7	28.3	>10	13.3	Taiwan
Mermel and Maki, 1993	< 5	20	–	–	USA
Chang et al., 2015	< 3	48.4	–	–	South Korea

a Data from 5 hospitals

b Thresholds were defined as 2 mL below and above the recommended volume per vial.

On the other hand, BC bottles are inoculated with more than 10 mL of blood in 7.6–13% of cases (Table 3). These bottles are at increased risk to be falsely flagged positive by the BC system (Wilson et al., 1994; Reimer et al., 1997). According to Willems et al. (2012), both Becton Dickinson®(BD) and bioMérieux® admitted that the vacuum in the BC bottles substantially exceeds the optimal blood fill volume (10 mL; Willems et al., 2012). The purpose of the vacuum excess is to ensure a sufficiently long expiration date, and to minimize the collection time. All these data underline how common are situations of non-compliance with manufacturer instructions, regarding the volume of blood to be inoculated for optimal diagnostic yield.

Secondly, BCs are commonly “solitary BC,” for various reasons (e.g., resolution of fever, venipuncture failures, patients discharged home, or to another department where the ordering of BCs is discontinued, omission in collecting subsequent BC sets). Several studies reported rates of solitary BC per center between 10 and 33.3% (Table 4). Proportions of solitary BC seem to be lower in the US, with medians of 26%, and 12.7% (Schifman et al., 1991; Novis et al., 2001), which may be related to the implementation of quality control programs that include this indicator, and to the fact that the personnel primarily responsible for BC collection are dedicated phlebotomists, two major differences with practices in most European countries. The combined effect of under-filled BC bottles and high proportion of solitary BCs, results in a high proportion of patients suspected of BSI for whom the total volume of blood inoculated is insufficient.

**Table 4:** **Rate of solitary blood cultures**.

**References**	**No. of institutions**	**Rate (%)**
Gross et al., 1988	1	28.0
Makadon et al., 1987	1	20.0
Schifman et al., 1991	38	26.0 (median)
Schifman et al., 1996	909	10.1–12.1 (inpatients) 25.4–33.3 (outpatients)
Novis et al., 2001	333	12.7 (median)
Vitrat-Hincky et al., 2011	1	28.0
Neves et al., 2015	1	23.2

Given the critical importance of sampling the adequate amount of blood for BC sensitivity, monitoring the volume of blood cultured is a strong quality-assurance requirement (Mermel and Maki, 1993; Schifman et al., 1996; Willems et al., 2012; Chang et al., 2015; Accoceberry et al., 2015a). It should be surveyed through systematic program, either internal or external to the microbiology lab, and should at least include the proportions of solitary BC, and of adequately filled BC bottles (Schifman et al., 1991, 1996; Mermel and Maki, 1993; Novis et al., 2001; Willems et al., 2012; van Ingen et al., 2013; Chang et al., 2015; Accoceberry et al., 2015a). This latter may be estimated through either measurement of the weight of BC bottles received at the laboratories or visual inspection, although the latter is less accurate.

### Sampling strategy

Sampling an adequate volume of blood to ensure optimal sensitivity for BSI detection can be achieved either by increasing the number of venipunctures (hereafter, “multi-sampling strategy”) or by collecting the adequate large volume through one single puncture (hereafter, “single-sampling strategy”).

#### Multi-sampling strategy

##### Rationale

The multi-sampling strategy has been developed, and recommended for more than 40 years, and its practice has been generalized (Washington, 1975, 1992). The rationale of this strategy is based on the following points: (i) repetition of samples increases the total volume of blood cultured, thereby improving BC sensitivity, (ii) separate samples may discriminate contaminants from pathogens when BCs grow, (iii) separate samples improve BSI detection in case of intermittent bacteremia (Washington, 1975; Reimer et al., 1997).

It has been successful to improve BSI detection in several studies which concluded that, under routine circumstances, at least two separate sets of BCs should be sampled during a 24-h period for the diagnosis of BSIs (Mermel and Maki, 1993; Li et al., 1994; Weinstein, 1996; Cockerill et al., 2004; Bouza et al., 2007; Lee et al., 2007), and guidelines recommend two to three - or four - BC sets (O'Grady et al., 2008; Lamy and Seifert, 2012; Baron et al., 2013; Dellinger et al., 2013; Public Health England, 2014; Accoceberry et al., 2015a).

##### Interpretation of positive culture results

The differentiation between clinically significant positive BCs (i.e., the micro-organism identified is involved in the symptoms presented by the patient), and contaminants, is based on the number of positive BC samples when the culture yielded an organism regarded as a common contaminant [e.g., coagulase-negative staphylococci (CoNS), *Corynebacterium* spp, *Micrococcus* spp, etc.] (MacGregor and Beaty, 1972). For these microorganisms, at least 2 positive BCs yielding the same CoNS are usually required to consider the results as clinically significant, given that the likelihood that it represents true bacteremia as opposed to contamination increases with the number of positive bottles (Weinstein, 1996; Reimer et al., 1997; Kirn and Weinstein, 2013; Garcia et al., 2015). This can assist in interpreting positive BC results, although the clinical context, including the presence of intravascular foreign devices, is also of paramount importance for an accurate interpretation of BCs positive for CoNS.

##### Limitations

Despite these recommendations, several issues have been highlighted: firstly, the proportion of solitary BCs is high with the multi-sampling strategy (Schifman et al., 1991; Vitrat-Hincky et al., 2011; Neves et al., 2015), and these are associated with a major default of sensitivity. In addition, solitary BC makes it more difficult to distinguish contaminants from pathogens. Secondly, each venipuncture required for the multisampling strategy is an additional opportunity for contamination (Aronson and Bor, 1987; Lamy et al., 2002; Patel et al., 2011). The contamination rate per draw has been estimated at 0.5–6% (Bates et al., 1991; Salluzzo and Reilly, 1991; Washington, 1992; Arendrup et al., 1996; Garcia et al., 2015). However, considering that only one positive sample may be interpreted as a confirmation that the patient has, indeed, BSI, and that the prevalence of BSI among patients suspected of BSI is relatively low, the proportion of false-positive among patients with positive BC may be as high as 20–56% (Bates et al., 1991; Salluzzo and Reilly, 1991; Little et al., 1997). The consequences of BC contamination and false positives, although poorly studied, are not trivial, as they may lead to longer hospital stays, useless prescription of antibacterial or antifungal agents, additional investigations (e.g., echocardiography, repeated sampling for BC; Bates et al., 1991; Souvenir et al., 1998; Hall and Lyman, 2006; Gander et al., 2009; Alahmadi et al., 2011). In a retrospective case-control study on 254 false-positive BC results, Alahmadi et al. (2011) demonstrated that hospital length of stay increased by 5.4 days (2.8–8.1), with an additional hospital cost of £1,270,381 per year. In another study, contaminated BCs increased patient charges by 47% with an estimated cost of $8,720 per contamination (Gander et al., 2009). Souvenir et al. (1998) reported that almost half of patients with positive BCs that were finally classified as “contaminants” were treated with antibiotics, including 34% by vancomycin. Additional costs were estimated at $1000 per patient in this study, with a median increase in length of stay of 4.5 days (Souvenir et al., 1998). False-positive BCs generated a 20% increase in laboratory tests and a 39% increase in intravenous antibiotic charges in another study (Bates et al., 1991).

Finally, the theoretical concept of intermittent bacteremia or fungemia that supports the multi-sampling strategy has never been proved. Evidence suggests that most cases of clinically significant BSI are associated with continuous bacteremia or fungemia over a 24 h period, but with very low concentrations of circulating microorganisms (Jonsson et al., 1993; Li et al., 1994; Riedel et al., 2008).

#### Single-sampling strategy

##### Rationale

The single-sampling strategy collects the total volume of blood from one single draw, a “BC set” of 4 to 6 bottles. This strategy satisfies both the need to collect a sufficient volume of blood, and the need to decrease contamination rate by limiting the number of punctures. In addition, this would be associated with decreased workload and risk of occupational exposure to blood-transmissible pathogens for nurses, decreased cost, and improved comfort for patients, by reducing the number of invasive, potentially painful, procedures. This strategy was developed since the late 1990s, based on the following: (i) the concept of intermittent bacteremia or fungemia may be erroneous (Jonsson et al., 1993; Li et al., 1994; Riedel et al., 2008); (ii) the key determinant for the capacity of BCs to diagnose BSI is the total volume of blood inoculated (Li et al., 1994); (iii) the rate of false-positive results increases with the number of draws; (iv) for a given volume of blood inoculated, the multi- and single-sampling strategies are expected to have similar sensitivity; (v) because the total volume is obtained at once with the single-sampling strategy, there is no risk of omitting subsequent draws, thereby eradicating the risk of solitary BC. Hence, the median total volume of blood inoculated will necessarily be greater; (vi) the single-sampling strategy should enable early initiation of empirical antibiotic treatment when indicated (e.g., severe sepsis), as there will be no need to postpone until subsequent sampling; and (vii) patient comfort will be improved, as only one venipuncture will be requested for this strategy.

**Figure 1:**
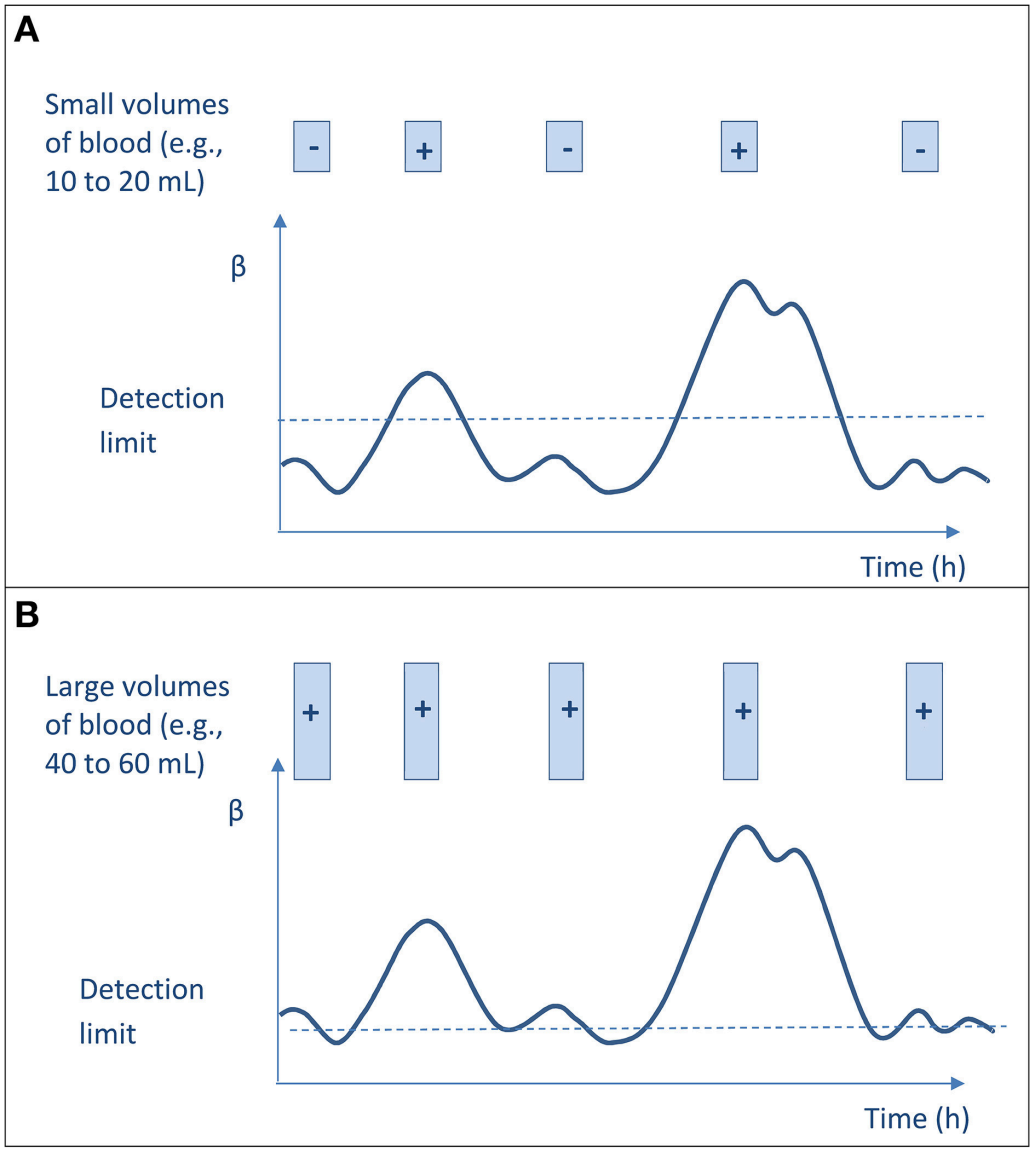
**Blood culture result (negative or positive) according to the amount of blood cultured at each sampling and to the microbial burden in blood**. The curve represents the bacterial concentration (β) in blood that varies with time and may be very low, but never null. The limit of bacteremia detection (BC sensitivity) is indicated with dotted line. Each sample is represented by a box. **(A)** Culturing low volumes of blood does not ensure sensitive testing and low detection threshold, and thus leads to uncertain bacteremia detection according to time of collection. The overall results suggest an intermittent bacteremia. **(B)** Culturing large volumes of blood ensures low detection threshold, thus allowing detecting bacteremia whenever the sample is obtained. One sample is enough for confidently detecting bacteremia; the overall results would suggest a continuous bacteremia.

First, as mentioned above, Li et al. (1994) demonstrated that increasing the volume of blood inoculated increases the yield of BC, whether or not BCs are drawn simultaneously or serially within 24 h (Li et al., 1994). To reconcile the interpretation of previous data (Washington, 1975; Cockerill et al., 2004) with these results, it has been hypothesized that the need to repeat BC sampling originated from the poor sensitivity when small volume of blood are inoculated (< 20 mL per set). In such a procedure, the detection depends on the bacterial density at time of samplings, and not all samples will turn positive. Hence, when large volume of blood is inoculated on BC media (i.e., 40 or 60 mL), the sensitivity of the single-sampling strategy is high whenever the sample is obtained and is similar to the sensitivity of multi-sampling strategy (Figure 1; Li et al., 1994; Lamy et al., 2002).

Arendrup et al. (1996) first evaluated the single-sampling strategy in their institution (Arendrup et al., 1996). Inoculating a single-sampling of 40-mL BC (4 bottles), instead of 30-mL BC (3 bottles) increased the diagnostic yield of BC by 4.2%. Using a before/after design, the authors concluded that this strategy had a positive impact on workload, and early initiation of empirical antimicrobial therapy. In most cases of positive BCs due to contaminants, only one or two of the four bottles turned positive, and the interpretation of positive BCs with a microorganism of questionable significance was not more difficult than with the multi-sampling strategy (Arendrup et al., 1996).

Two studies evaluated the impact of the number of draws on the risk of false-positive BCs (specificity), and quantified it using theoretical probabilistic approach (Aronson and Bor, 1987; Lamy et al., 2002). Both confirmed the negative impact of the number of separate sampling on BC specificity. One model-based study compared the performance (sensitivity and specificity) of the multi-sampling vs. the single-sampling strategies, using literature-based simulations and a quantitative risk-analysis approach. The median specificity of positive BCs decreased from 0.98 with the single-sampling strategy, to 0.91 with the 3 sample-strategy, which resulted, with a pretest probability of BSI set at 15%, in a median positive predictive value decrease from 0.85 to 0.66, respectively (Lamy et al., 2002). On the opposite, a 6 bottle-collection of blood at once (totalizing 35–42 mL) ensured an efficient diagnosis of bacterial BSI with an optimized specificity (Lamy et al., 2002). In this scenario, with a median volume of 7 mL of blood per bottle, the sensitivity was ≥0.95 in 89% of the trials, and the median specificity was 97.5%. Recently, a prospective study performed in the adult emergency departments of three university hospitals compared a single-sampling strategy collecting one large volume of blood (4 bottles), to the standard multi-sampling strategy. Overall, the single-sampling strategy allowed detection of pathogens in blood of 97.4% of patients vs. 95.5% for the multi-sampling strategy. In the subgroup of patients for whom two sets were drawn, the single-sampling strategy was superior to the multi-sampling strategy in term of positive results. In addition, considering the overall performances (sensitivity and specificity), the single-sampling strategy was significantly better than the multi-sampling strategy (Dargère et al., 2014). Finally, as expected, the proportion of solitary BC rate was strikingly reduced by using the single-sampling strategy.

Although this strategy has been approved in France as an alternative to the multi-sampling strategy since 2007 (SFM, 2007) a nationwide survey conducted in 47 hospitals found that only 17 and 21% of them were using the single-sampling strategy in 2013, exclusively and partly, respectively (Royer et al., 2015). In Denmark, where the single-sampling strategy first spread, a survey on BC practices was conducted in 2015 among 6/11 departments of clinical microbiology, covering half of Danish hospital admissions (1.28/2.57 millions admission). Responses showed that 1 out of 6 departments practiced the multi-sampling strategy, 2 practiced either multi-sampling or single-sampling strategy, and 3 hospitals practiced the single-sampling strategy. Five of these centers use the multi-sampling strategy when endocarditis is suspected.

##### Interpretation of positive BC results

Rules for interpretation of positive BC results, and distinction between BSI and contamination, differ with the single-sampling strategy, as the information given by the proportion of positive BC sets at different times cannot be used. In order to define guideline to differentiate clinically significant bacteria from contaminants with this strategy, a study was conducted between 2007 and 2008 in Lyon University Hospital (France), where the single-sampling strategy has been implemented in 2004 (Leyssene et al., 2011). In monomicrobial positive sets (one set being defined as 6 bottles originating from a single venipuncture), the positive predictive value (PPV) of BC was 88 and 100% with, respectively, one and ≥2 positive bottles for *E. coli*, while it was 100% for *S. aureus, Pseudomonas aeruginosa*, and *Candida* spp., whatever the number of positive bottles. For CoNS, the PPV with one, two, three or ≥4 positive bottle(s) was 3.5, 61.1, 78.9, and 100%, respectively. The most difficult cases to interpret were those with 2 or 3 positive bottles out of the 6 cultured, but this was the case for only 5% of patients with CoNS-growing BC. Therefore, despite systematic evaluations of an approach based on the number of positive bottles have proven to be unreliable when using the multi-sampling strategy (Mirrett et al., 2001b; Kirn and Weinstein, 2013), the clinical significance of CoNS-growing BC was correlated with the number of positive bottles when using the single-sampling strategy (Leyssene et al., 2011). Similar findings were observed in Arendrup et al. study (Arendrup et al., 1996). Consistent with these data, Dargère et al. (2014) showed that most contaminants were detected only in the first aerobic bottle of the 4-bottle set with the single-sampling strategy, and that the most frequent microorganism was CoNS (Dargère et al., 2014).

##### Limitations

Firstly, getting a sufficient volume of blood to fill 6 bottles from a single venipuncture may be difficult, particularly in the elderly, and in patients with shock. This may require an additional puncture to collect the total volume of blood necessary for optimal diagnostic yield of BCs. Secondly, the level of evidence on the performance of the single-sampling strategy remains low: only two published studies evaluated the performances of a single-sampling strategy based on inoculating 40 mL of blood (Arendrup et al., 1996; Dargère et al., 2014). The first one was a quasi-experimental study (i.e., before/after design), and the second one was randomized, but limited by sample size (*n* = 2374). Indeed, this lack of studies is mainly due to the fact that comparative studies are difficult to perform as they require a complex design to prevent biases, large sample sizes to be adequately powered, and a strict methodology to control the volume of blood actually cultured in each group. Thirdly, level of evidence is also lacking for specific situations such as infective endocarditis (IE)—for which the number of positive samples on distinct venipunctures is part of the modified Duke Criteria (Li et al., 2000)—or for CLABSI. A high BC sensitivity is expected for detecting IE with the single-sampling strategy because of the high density of bacteria in blood (Werner et al., 1967), but modified Duke Criteria would require to be adapted if the single-sampling strategy was to be generalized. In patients suspected of CLABSI, the diagnosis may be obtained through a single-sampling strategy (4–6 bottles) obtained through venipuncture, associated with one appropriately filled bottle simultaneously drawn from the catheter line to be able to estimate the differential time to positive BC (DTTP). Indeed, despite one peripheral BC set has been shown to be appropriate for the DTTP-based diagnosis of CLABSI (Mermel et al., 2009; Guembe et al., 2012), a 4–6 bottle set drawn by venipuncture is still indicated for the diagnosis of BSI, whatever the source, including thus BSI other than CLABSI. However, such protocols need to be investigated.

In addition, to the best of our knowledge, the medico-economic aspects of these two strategies have not been thoroughly evaluated. Other potential impacts of the single-sampling strategy remain to be measured, including patient comfort (e.g., only one venipuncture with the single-sampling strategy), the risk of occupational-exposure to blood-transmissible pathogens, and the timing of empirical anti-infective agent initiation.

## Blood culture systems

In this review, considerations on BC systems will be limited to those that may impact the performance (sensitivity, specificity) of BSI diagnosis. Overall, three continuous-monitoring automated BC systems are available in 2015. The two system most commonly used are, by far, the Bactec (Beckton-Dickinson, USA) and BactAlert/Virtuo systems (bioMérieux, France). These two systems are based on the utilization of carbohydrate substrates in culture media, and subsequent production of CO_2_ by growing microorganisms, detected through their impact on pH, by either a colorimetric, or a fluorescent sensor placed at the bottom of the bottle. Both systems have similar characteristics, and, despite differences in culture media, they share similar performances for detection of microorganisms (Kirn and Weinstein, 2013). The fertility of the culture media used in these systems is among the highest among broth media available in clinical microbiology. Media are combined in sets in order to include aerobic and anaerobic formulations, and those associated with resins or charcoal regularly show better performances as compared to standard media (Weinstein et al., 1995; Wilson et al., 1995, 2001; Gibb et al., 1998; Mirrett et al., 2004). Only slight improvements in the performances have been achieved over the last 15 years (e.g., Kirn et al., 2014).

Combined with inadequate sampling volumes, the early use of antibiotics prior to the first blood sample is a critical factor hampering microorganism recovery. Some media include resin or charcoal to neutralize antibiotics. The superior neutralizing capacity of resin-based media over the bioMérieux charcoal medium has been demonstrated (Flayhart et al., 2007), but discrepancies have been reported and the complete neutralization might be insufficient. The types and compositions of resins, the type of antibiotics and the MICs of the test strains may account for the differences in neutralization performance (Mitteregger et al., 2013). As far as possible, BCs should be obtained before the antimicrobial therapy is initiated.

### False-positive instrument signal

False-positive instrument signal, defined as a bottle flagged positive by the system though not containing any micro-organism, has been described with the systems, in less than 1% of bottles (Pohlman et al., 1995; Mirrett et al., 2001a). These false-positive bottles require quick handle and re-incubation into the BC system (e.g., within 1 h following their unloading) in order to resume the bottle analysis. Major causes of false-positive results include high level of leukocyte counts, over-filled bottles and/or errors in incubation so that the bottles are monitored with an inappropriate algorithm (Wilson et al., 1994; Reimer et al., 1997). However, available data are scarce and mostly derive from studies of media that are not in use anymore. Given the significant changes in media formulation over years, updated evaluations are required to evaluate the actual incidence of false-positive signals, and the effect of over-filled bottles on positivity.

### False-negative instrument signal

False-negative instrument signal is defined as a bottle flagged negative by the system although it contains bacteria or fungi. The main parameter associated with false-negative signal is the pre-incubation delay, which is the time difference between the time point of bottle loading in the BC incubator and the time point of bottles filling. It has been estimated that 0.3 to 15.3% of bottles containing bacteria or fungi are flagged negative by BC system (Reimer et al., 1997; Klaerner et al., 2000; Mirrett et al., 2001a; Lemming et al., 2004; Seegmüller et al., 2004), although few of the media currently in use have been evaluated for this default. The false-negative signal depends on the temperature at which the bottles were kept before loading (hereafter pre-incubation temperature), the pre-incubation duration, the type of micro-organism involved, and the pairing instrument/type of bottle used. Overall, the false negative signal rate was greater when pre-incubation temperature was 35°C, pre-incubation duration was > 24 h, with streptococci, *Candida* sp, or *Pseudomonas* sp, and with Bactec systems (Sautter et al., 2006). A pre-incubation duration < 12 h kept the risk of false negative at its minimum (Lemming et al., 2004; Seegmüller et al., 2004; Akan and Yildiz, 2006; Sautter et al., 2006). It has been hypothesized that micro-organisms grow in the bottle during the pre-incubation duration, especially with high temperature (35°C), so that they have already reached the stationary phase at time of loading. In such a situation, positive bottles cannot be detected by the instrument algorithms. Only 60% of false-negative signals were detected by visual inspection before loading (Lemming et al., 2004). Pre-incubation at 35°C should be discouraged (Lamy and Seifert, 2012; Baron et al., 2013; Kirn and Weinstein, 2013; Accoceberry et al., 2015a), and suppliers have revised their guidelines in this way. Indeed, bottles should be delivered to the laboratory and loaded in the BC system as fast as possible (Seegmüller et al., 2004). This point is pivotal especially as studies on BC transport time have shown that delayed entry negatively impacts on time to positivity from the time point of sampling (Ronnberg et al., 2013) or on the positive BC rate (Morton et al., 2015). Indeed, Morton et al. (2015) highlighted that BC yield was lower when BCs were collected during the week-end, and this was likely caused by delays or errors in incubation and processing, in relation with the reduced provision of support services during the week-ends (Morton et al., 2015).

### Monitoring the volume of blood inoculated

As underlined, a major cause for missing a diagnosis of BSI is insufficient filling of BC bottles, which leads to a sub-optimal sensitivity. Improvement of BCs bottle filling would require enhanced training of phlebotomists, including systematic visual inspection of bottle filling at the bedside (van Ingen et al., 2013). In addition, some BC system manufacturers developed automated systems to estimate the level of bottle filling at the time of loading (Chang et al., 2015). The Bactec FX system (BD, USA), based on red blood cells metabolism, provides reliable estimates of the blood volume in BC bottles, with a mean error of 0.2 mL. The error margin is higher (i.e., 1 mL), for BC bottles received from hematology wards, due to anemia or impaired red blood cells metabolism (Chang et al., 2015). One limitation of this device is that volume is monitored in batches and not for each bottle individually. A device developed on the new BC system Virtuo (bioMerieux) provides an automated volume estimation of each bottle based on a photometric detection of liquid level, but, as far as we know, its accuracy has not yet been evaluated in clinical studies.

### Incubation time

Bottles are usually incubated for a maximum duration of 5–7 days (Lamy and Seifert, 2012; Baron et al., 2013; Kirn and Weinstein, 2013; Accoceberry et al., 2015a). Very few micro-organisms are recovered between 5 and 7 days. Among these, clinically significant bacteria or fungi are mostly low-level CO_2_ producers, or slowly growing micro-organisms (e.g., *Candida* spp, strict anaerobes, or *Brucella* sp). Marginson et al. (2014) found that 2.7% of all positive BC turned positive between 5 and 7 days, including 0.5% of clinically significant micro-organism (Marginson et al., 2014). When infective endocarditis is suspected but BC remain negative on day 5, some guidelines state that bottles incubation must be prolonged until day 15 or more (Mainardi and Ricardo, 2012; Accoceberry et al., 2015b). However, these recommendations are mostly based on data originating from studies performed before the use of automated BC systems and highly-efficient culture media (Petti et al., 2006). Studies based on continuous-monitoring BC systems have shown that prolonged incubation time does not significantly improve the overall sensitivity of BC, even for IE due to fastidious bacteria from the HACEK group (*Haemophilus, Aggregibacter, Cardiobacterium, Eikenella, Kingella*) as all of these were recovered from a standard 5-day incubation protocol in various studies (Wilson et al., 1993; Baron et al., 2005a; Petti et al., 2006). Prolonged cultures of specific BC bottles for the detection of fastidious zoonotic agents in blood culture-negative endocarditis (BCNE) are exceptionally useful, and are not recommended in routine practice. Fournier et al. (2010) have demonstrated that serological analysis was the most useful specimen providing a diagnosis of 47.7% of 745 tested patients with suspected BCNE (mainly Q fever and *Bartonella* infections; Fournier et al., 2010). According to the local epidemiology, the diagnosis of BCNE is based on systematic serological testing for *Coxiella burnetii, Bartonella* spp., *Brucella* spp., *Aspergillus* spp., *Mycoplasma pneumonia, Legionella pneumophila* followed by specific polymerase chain reaction (PCR) assays (*Tropheryma whipplei, Bartonella* spp. and fungi) (Baddour et al., 2015; Habib et al., 2015).

## Conclusions

BCs are among the most common microbiological tests performed in 2016 and remain the first and essential diagnostic tool for detection of BSIs. Best practices of BC sampling require thorough understanding of several issues including appropriate ordering BCs, timing of BC collection, skin preparation, sample site, impact of the volume sampled. Quality control programs, including automated controls of pre-analytical variables should be reinforced to address the deficiencies that are frequently reported.

While both sampling strategies (i.e., multi-sampling strategy, or single-sampling strategy) are acceptable according to the guidelines, additional progresses are expected with the single-sampling strategy that is probably more convenient for patients and healthcare workers and probably associated with reduced costs, earlier initiation of empirical anti-infective treatment when indicated, and lower risk of occupational exposure to blood-transmissible pathogens. Today, evidence-based clinical data to document the yield of this strategy deserve to be improved and a large multicenter randomized trial is required to decipher the pro and cons of both strategies of BC collection for the diagnosis of BSIs and infective endocarditis.

## Author contributions

Conceived and designed the work: JJP, SD, BL, and PT. Performed survey: MC. Drafted the paper: BL, SD, and PT. Critically revised the manuscript: PT, MA. All authors read and approved the final manuscript.

### Conflict of interest statement

The authors declare that the research was conducted in the absence of any commercial or financial relationships that could be construed as a potential conflict of interest.
